# Determinants of price setting decisions on anti-malarial drugs at retail shops in Cambodia

**DOI:** 10.1186/s12936-015-0737-9

**Published:** 2015-05-30

**Authors:** Edith Patouillard, Kara Hanson, Immo Kleinschmidt, Benjamin Palafox, Sarah Tougher, Sochea Pok, Kate O’Connell, Catherine Goodman

**Affiliations:** London School of Hygiene and Tropical Medicine, 15-17 Tavistock Place, London, WC1H 9SH UK; Population Services International Cambodia, No. 29 Street 334, P.O. Box 153, BKK1 Chamcar Mon Phnom Penh, Kingdom of Cambodia; Malaria and Child Survival Department, Population Services International (PSI), PO Box 14355–00800, Nairobi, Kenya

**Keywords:** Price setting decisions, Retail sector, Malaria treatment, Drug resistance

## Abstract

**Background:**

In many low-income countries, the private commercial sector plays an important role in the provision of malaria treatment. However, the quality of care it provides is often poor, with artemisinin combination therapy (ACT) generally being too costly for consumers. Decreasing ACT prices is critical for improving private sector treatment outcomes and reducing the spread of artemisinin resistance. Yet limited evidence exists on the factors influencing retailers’ pricing decisions. This study investigates the determinants of price mark-ups on anti-malarial drugs in retail outlets in Cambodia.

**Methods:**

Taking an economics perspective, the study tests the hypothesis that the structure of the anti-malarial market determines the way providers set their prices. Providers facing weak competition are hypothesized to apply high mark-ups and set prices above the competitive level. To analyse the relationship between market competition and provider pricing, the study used cross-sectional data from retail outlets selling anti-malarial drugs, including outlet characteristics data (e.g. outlet type, anti-malarial sales volumes), range of anti-malarial drugs stocked (e.g. dosage form, brand status) and purchase and selling prices. Market concentration, a measure of the level of market competition, was estimated using sales volume data. Market accessibility was defined based on travel time to the closest main commercial area. Percent mark-ups were calculated using price data. The relationship between mark-ups and market concentration was explored using regression analysis.

**Results:**

The anti-malarial market was on average highly concentrated, suggesting weak competition. Higher concentration was positively associated with higher mark-ups in moderately accessible markets only, with no significant relationship or a negative relationship in other markets. Other determinants of pricing included anti-malarial brand status and generic type, with higher mark-ups on cheaper products.

**Conclusions:**

The results indicate that provider pricing as well as other key elements of anti-malarial supply and demand may have played an important role in the limited access to appropriate malaria treatment in Cambodia. The potential for an ACT price subsidy at manufacturer level combined with effective communications directed at consumers and supportive private sector regulation should be explored to improve access to quality malaria treatment in Cambodia.

**Electronic supplementary material:**

The online version of this article (doi:10.1186/s12936-015-0737-9) contains supplementary material, which is available to authorized users.

## Background

In many low-income countries, private commercial providers play an important role in the provision of malaria treatment [1–6]. Patient choice of private over public sector providers is influenced by several factors including the wider range of drugs stocked in private outlets, proximity to patients’ homes, less frequent stock outs and greater responsiveness to consumer preferences. However, the quality of care they provide is often poor, with commercial providers often lacking relevant qualifications and adequate knowledge of malaria national treatment guidelines [7, 8].

One of the cornerstones of malaria control is parasitological confirmation of all suspected malaria cases by either microscopy or rapid diagnostic tests (RDTs) and treatment of confirmed *Plasmodium falciparum* cases only with artemisinin-based combination therapy (ACT). By 2009, most countries with *P. falciparum* had switched to ACT as their first-line medicine, with the choice of combination drugs based on their efficacy in specific countries.

The therapeutic life of ACT is, however, threatened by resistance to artemisinins confirmed on the Cambodia-Thailand border and more recently detected in two other countries in the Greater Mekong subregion, Myanmar and Vietnam [9]. This is of major concern to the international community, particularly as resistance may spread to countries with much higher disease burdens in Africa, as has been the case in the past for older anti-malarial drugs [10–14]. The loss of ACT to resistance would be catastrophic for malaria control strategies as no other treatment with the same efficacy and tolerability is currently available [15].

Containing resistance to areas where it exists is, therefore, paramount. Factors believed to encourage the emergence and spread of anti-malarial resistance include lack of confirmed diagnosis through blood testing, the consumption of oral artemisinin monotherapies (AMT), poor consumer adherence to treatment regimens and poor quality drugs [16, 17]. Increasing access to parasitological diagnosis of all cases with suspected malaria and rational treatment with affordable ACT for *P. falciparum* confirmed cases, including in the private sector, are key recommended responses for the containment of artemisinin resistance [9]. Complementary actions include the removal of oral AMT and substandard and counterfeit drugs from the market, and educative communication campaigns targeting consumers and providers [18].

Cambodia finds itself at the heart of these issues, given its malaria burden, resistance profile and large private sector. Malaria transmission is seasonal (from May to December) and is concentrated in forested areas. Around 15 % of the population (2.2 m people) is at risk of infection, predominantly male adults. The national malaria treatment policy specifies that all people with suspected malaria should first receive a blood test, either using microscopy or RDTs [19]. At the time of the study in 2009, first-line treatment of confirmed uncomplicated adult *P. falciparum* cases consisted of the ACT artesunate and mefloquine (ASMQ) for three days while first-line treatment of *Plasmodium vivax* cases was chloroquine, also for three days [19]. The sale and distribution of oral AMT had been prohibited. In 2009, the country could be roughly divided into two strata: the Western part where resistance was confirmed or suspected, and the North and Northeast, where *P. falciparum* remained susceptible to ACT.

In 2009, the commercial sector supplied about 75 % of all malaria treatments in Cambodia [20, 21]. Commercial anti-malarial providers included pharmacies or clinical pharmacies, drug shops, mobile providers, grocery stores and village shops. ACT and RDTs have been the focus of a social marketing programme since 2002, which includes consumer behaviour change communication, provider training, and the sale of subsidized ACT and RDTs. In 2009, one adult pack of ACT was sold to commercial providers for US$0.42 with a recommended retail consumer price (RRP) of US$0.61 printed on each pack. In the public sector, parasitological diagnosis and anti-malarials were available free of charge from government-owned outlets, including health centres and hospitals and a network of trained volunteers (referred to as Village Malaria Workers (VMWs)) [20] in areas with high malaria risk and with no access to health facilities.

Despite these initiatives, evidence shows that in 2009 clinical practices rarely followed national treatment guidelines and that the quality of fever management was often low both at public and private outlets [20]. Overall, only 47 % of positive malaria cases received an anti-malarial and 35 % an ACT [22] whilst AMT, which are banned, accounted for 15 % of all anti-malarials dispensed [20]. “Cocktail therapies”, a mix of several different drugs, were the most common treatment for malaria fevers received in the commercial sector, with 47 % of respondents receiving a cocktail containing no anti-malarial and 11 % a cocktail with an anti-malarial drug [20]. These poor treatment practices are related to ACT availability and consumers’ perceptions of product quality. Whilst knowledge about the recommended first line treatment for *P. falciparum* was generally high amongst anti-malarial drug sellers [20], ACT availability was low, ranging between 1 % and 49 % across different commercial provider types [20]. It has been reported that provision of cocktail treatments may be perceived by consumers as reflecting provider knowledge and expertise [21, 23] whilst oral AMT may be seen as causing fewer side effects than the ACT ASMQ [21, 24].

Despite the subsidy programme, retail price is also still likely to have been an important determinant of ACT uptake. For instance, in 2009 the median retail price of one ACT adult equivalent treatment dose (AETD) was US$1.18, roughly two times the RRP. The structure of the market for anti-malarial drugs is likely to have an important impact on private sector prices. A market is commonly defined as the set of sellers and buyers of a good or service whose interactions determine the quantity, quality and price of the good or service [25]. Market structure is defined as the characteristics of the market, including the range of sellers and products available, the number of sellers and their relative importance in terms of sales volumes and values (also referred as the degree of market concentration), the presence and intensity of barriers to market entry and exit, the structure of the retail distribution chain and the regulatory system. The structure-conduct-performance (SCP) framework predicts that the structure of the market determines the way firms behave which, in turn, affects market performance [26]. In particular, providers facing weak competition may have the opportunity to raise their prices above levels seen in more competitive markets. Weak competition is traditionally characterized by more concentrated markets, where fewer providers account for larger market shares [27]. This is compounded if there is limited competition from potential new providers (termed limited contestability), due to high barriers to entering the market such as costly retail outlet license fees.

Few studies have investigated the association between market structure and price setting decisions in health care markets in low-income countries [28–33]. One study in rural Tanzania found that higher prices were statistically associated with higher market concentration [32]. The aim of this paper is to analyse the determinants of retailers’ price setting decisions for anti-malarial drugs in Cambodia, and draw policy recommendations for improved access to affordable quality malaria treatment and resistance containment.

## Methods

### Study design

The study draws on cross-sectional survey data from anti-malarial outlets to define the boundaries of the markets under study, calculate market concentration, measure pricing using price or price mark-up data, and assess the association between pricing and market concentration through regression analysis. The regression analysis controls for cost and quality variations that may also affect pricing using market, outlet and product characteristics. Product-level variables include anti-malarial generic type, dosage form and brand status, while outlet-level variables are outlet type, outlet location (drug resistance stratum), number of years in operation, anti-malarial volume sold during the week preceding the survey and wholesaler delivery practices. Market-level variables include accessibility and malaria transmission risk. For example, in more remote markets, it is likely to be more costly to obtain drug supplies (e.g. due to transport costs) putting upward pressure on prices. In markets with higher risk of malaria transmission, competition from government providers, notably VMWs who give free treatment and are physically more accessible, may be more intense than in areas at lower risk, constraining commercial providers’ pricing. Markets in areas of high malaria transmission could also be more contestable (with lower entry barriers), contributing to lower commercial prices.

### Data sources

Data from the 2009 Cambodia ACTwatch outlet survey were used. The ACTwatch outlet survey is a nationally representative cross-sectional study conducted in a sample of outlets in 38 sub-districts. A sub-district is the catchment area of a government health centre, which provides primary health care services to around 10,000 inhabitants. Details on the outlet survey design and survey procedures are published elsewhere [20]. Briefly, the sample of 38 sub-districts was stratified by drug resistance level, with 19 sub-districts sampled from areas with suspected or confirmed artemisinin resistance and 19 from areas with no artemisinin resistance. In each stratum, the 19 sub-districts were sampled using probability proportional to population size. In each sampled sub-district, a census of all public and private outlets that stocked anti-malarial drugs was conducted. Data collected included outlet characteristics, anti-malarial drugs stocked, and, for each anti-malarial stocked, purchase and selling prices and recall of volumes sold over the previous week. Data on cocktails were excluded as it was not possible to assess their content and therefore whether they contained an anti-malarial, and if they did which one. Census data on outlet characteristics and range of anti-malarial drugs stocked were used to define the markets for malaria treatment (as described below), and to control for product quality and cost variations in the analysis of providers’ price setting decisions. Sales volume data were used to measure market concentration. Market level variables (accessibility and transmission risk) were drawn from the 2008 Cambodia national census data, National Establishment Listing, Ministry of Health (MOH) risk categorization of malaria endemic areas, and interviews with key stakeholders.

### Defining markets

The literature conventionally defines markets along geographic and product lines. The product definition was set as all anti-malarial drug types stocked by all outlets selling anti-malarials based on census data from the ACTwatch outlet survey [20]. Informed by qualitative interviews with anti-malaria providers on the provenance of anti-malarial customers and location of competitors, the geographic definition was set as the commune, an administrative unit of around 50 km^2^ and about 3500 inhabitants. A total of 87 geographic markets were defined.

### Measuring market concentration

Market concentration was measured using the Herfindahl-Hirschman-Index (HHI), which is calculated as the sum of the squares of the market shares of all providers operating in the market. US anti-trust agencies generally classify markets into three types depending on the HHI value: (1) unconcentrated markets with HHI below 0.15 (2) moderately concentrated markets with HHI between 0.15 and 0.25 or (3) highly concentrated markets with HHI above 0.25 [34]. Market concentration was calculated in terms of the volume of anti-malarial sales, using data on all anti-malarials stocked at each anti-malarial outlet surveyed. Market concentration was also calculated in terms of the value of anti-malarial sales. As it did not make a difference in the study results, the paper focuses on the analysis conducted using market concentration calculated on anti-malarial sales volumes. Both private and public anti-malarial sales were included in the calculations, with all government outlets within each market treated as one provider on the basis that government providers were not expected to compete with one another. Because of variation in dosage and formulations across different anti-malarials, sales volume data were calculated in terms of adult equivalent treatment doses (AETDs), defined as the number of milligrams of an anti-malarial drug needed to treat a 60 kilogramme adult [20]. Missing sales volume data (around 14 % of all sale volume data) were imputed using the mean matching multiple imputation method in Stata 11 [35]. The method uses a standard linear regression of sales volumes on a set of explanatory variables to obtain predictions. Variables hypothesized to explain most of the variation in sales volumes across outlet and anti-malarial types included geographical location, anti-malarial category, brand name, generic type, dosage form and manufacturer [36].

### Estimating market accessibility and malaria transmission risk levels

Accessibility was measured as the time required to travel in a 4-wheel drive vehicle from each commune to the main city of the closest main commercial area, defined as the closest province with more than 5 % of all commercial establishments in the country. Markets were grouped into 3 categories: “accessible” (markets located less than 2.5 h from the closest commercial area); “moderately accessible” (2.5–4.5 h); and “remote” (more than 4.5 h). Following the MOH malaria transmission risk categorization, markets were classified at “high risk” of malaria transmission if they were located less than 250 m from the forest, at “moderate risk” if located between 250 m and less than 1 km from the forest, and at “low risk” if located 1 km or more from the forest. For markets that covered areas with different risk levels, the number of people living in the different risk level categories was calculated using 2008 census data [37] and markets were assigned the level of risk that was most common in that area in terms of number of inhabitants exposed.

### Modelling price setting decisions

At each commercial retail outlet and for each anti-malarial product stocked, purchase and selling prices were calculated per AETD. For each product, the percent mark-up was calculated as the difference between selling price and purchase price, divided by purchase price and multiplied by 100. Percent mark-ups were preferred to absolute mark-ups (the difference between selling and purchase prices) as they offered a measure of retailers’ decisions “standardized” by price level. Price and mark-up data were summarized by calculating the median and inter-quartile range.

In the regression models, percent mark-ups were used as the dependent variable. Mark-ups were modelled rather than retail prices as to a large degree retail prices are determined by factors at the manufacture and wholesale levels, which are beyond the control of retail providers and therefore not expected to be affected by local retail market conditions.

Percent mark-up data from commercial retail outlets were analysed for all generic types and formulations of anti-malarials with more than one observation available. Percent mark-ups were log transformed because of their skewed distribution and regressed using Ordinary Least Square (OLS) on a set of market, outlet and product characteristics (Table 1). To present the effect of a change in a dependent variable on mark-ups rather than on log mark-ups, coefficients were back-transformed by calculating their exponent.

**Table 1:** Description of variables used in the model of anti-malarial price mark-ups

Variables	Definition	Mean
Log Price Mark-Ups	Log of retail percent price mark-up on one adult equivalent treatment dose	3.81
Market characteristics		
Concentration	Hirschman Herfindahl index on private and public sector anti-malarial sales volumes by market^a^	0.33
High malaria risk (reference)	1 if market is at high malaria transmission risk	0.23
Moderate malaria risk	1 if market is at moderate malaria transmission risk	0.39
Low malaria risk	1 if market is at low malaria transmission risk	0.38
Remote (reference)	1 if market is remote (more than 4.5 h drive to closest commercial centre)	0.23
Moderately accessible	1 if market is moderately accessible (between 2.5 and 4.5 h drive)	0.43
Highly accessible	1 if market is accessible (less than 2.5 h drive)	0.33
Outlet characteristics		
Located in areas without drug resistance (reference)	1 if area is without drug resistance	0.39
Located in areas with suspected/confirmed drug resistance	1 if area with resistance suspected or confirmed	0.61
Pharmacies/Clinical Pharmacies (reference)	1 if outlet is pharmacy/clinical pharmacy	0.24
Drug Shop	1 if outlet is drug store	0.22
Mobile Provider	1 if outlet is mobile provider	0.24
Grocery Store	1 if outlet grocery store	0.13
Village Shop	1 if outlet is village shop	0.16
Collects supplies from wholesaler	1 if none of the wholesalers deliver supplies to outlet	0.59
Receives supplies delivered by wholesalers	1 if at least one top supplier delivers	0.41
No years in operation	Number of years outlet has been in operation	10.7
No AETD sold in past 1 week	Number of adult equivalent treatment doses of anti-malarials sold in the previous week	2.19
Product characteristics		
Tablet (reference)	1 if anti-malarial is in tablet form	0.89
Injectable	1 if anti-malarial is in injectable form	0.11
Not branded (reference)	1 if anti-malarial is unbranded generic	0.73
Branded	1 if anti-malarial is branded innovator or generic	0.27
Artesunate + Mefloquine (reference)	1 if anti-malarial is ACT artesunate + mefloquine	0.49
Artemether	1 is anti-malarial is artemether	0.06
Artesunate	1 if anti-malarial is artesunate	0.15
Chloroquine	1 if anti-malarial is chloroquine	0.07
Dihydroartemisinin	1 if anti-malarial is dihydroartemisinin	0.01
Dihydroartemisinin + Piperaquine	1 if anti-malarial is dihydroartemisin + piperaquine	0.05
Mefloquine	1 if anti-malarial is mefloquine	0.01
Quinine	1 if anti-malarial is quinine	0.12
Sulfadoxine-Pyrimethamine	1 if anti-malarial is sulphadoxine-pyrimethamine	0.24
Dihydroartemisinin + Piperaquine + Primaquine	1 if anti-malarial is ACT dihydroartemisin + piperaquine + primaquine	0.03

^a^geographical definition of retail markets was set as the commune

Correlation coefficients between the variables used in our model are presented in Additional file 1. The Stata survey estimation command svy:regress was used to adjust for potential clustering of drug price observations within outlets, to control for design-based heteroscedasticity and to produce robust variance estimates (StataCorp [38]). Markets with a single anti-malarial outlet were treated as single sampling units with their variance imputed using the average of the variances from strata with several anti-malarial outlets (StataCorp [38]).

Sales, price and mark-up data analyses used weights to account for the outlet survey sampling strategy, which included a census of commercial and government outlets in sub-districts of varying size selected using probability proportional to size, and variation in the size of the strata. Sub-district weights, which were weights equal to the inverse of the probability that a sub-district was selected, were applied during data analysis using the Stata commands aweight or svyset depending on the calculations performed [20].

A first model (Model 1) of percent mark-ups was developed, as below, where ε is the error term:

$$ \begin{array}{l} \log mark- up={\beta}_0 + {\beta}_1HHI+{\beta}_2\  stratum+{\beta}_3 accessibility+{\beta}_4 risk+{\beta}_5 outlet\  type+\\ {}{\beta}_6 supplier\  delivers + {\beta}_7 time\  in\  operation+{\beta}_8 generic\  type+{\beta}_9 brand\  status+\\ {}{\beta}_{10} dosage\  form+{\beta}_{11} volumes\  sold+\varepsilon \end{array} $$

It is possible that a change in concentration may have affected mark-ups differently in markets with different characteristics, including accessibility or malaria transmission risk levels. Therefore a second model (Model 2) was estimated to explore the effect of interactions between market concentration, accessibility and risk on percent mark-ups:

$$ \begin{array}{l} \log mark- up={\beta}_0 + {\beta}_1HHI+{\beta}_2\  stratum+{\beta}_3 accessibility+{\beta}_4 risk+{\beta}_5\ HHI* accessibility + \\ {}{\beta}_6HHI* risk + {\beta}_7 outlet\  type+{\beta}_8 supplier\  delivers + {\beta}_9 time\  in\  operation+\\ {}{\beta}_{10} generic\  type + {\beta}_{11} brand\  status+{\beta}_{12} dosage\  form+{\beta}_{13} volumes\  sold+\varepsilon\;\end{array} $$

All interaction terms were included at once, and the statistical significance of their effect on retail percent mark-ups tested using the adjusted Wald test. Where the effect of an interaction term was not statistically significant, it was dropped and the model re-run to assess the effect on the remaining interaction terms and other estimates. Insignificant interaction terms were dropped one at a time and the process repeated until the best fitting model was identified. Coefficients and 95 % CI of linear combinations of predictor variables were calculated using the Stata post-estimation command lincom [38].

## Results

### Market structure

The number of anti-malarial providers was very variable across the 87 markets, ranging from 1 to 32, with a mean of 5 per market. In 61 % of the markets, both government and commercial providers dispensed anti-malarials, while in 30 % there were only commercial providers, and in 9 % only VMWs. Market concentration was measured for 73 of the 87 markets as in 14 markets total sales volumes were zero. The median HHI was 0.50 (IQR 0.34-0.74), which indicates high concentration according to US anti-trust guidelines [34]. Around 43 % of markets were classified as remote, 32 % as moderately accessible and 25 % as accessible. In terms of malaria transmission risk, 51 % of markets were at moderate risk, 31 % at low risk and 18 % at high risk. Accessible markets were roughly equally distributed across the three malaria transmission risk levels. By contrast, significantly more remote markets were at moderate risk of malaria (73 %) than at low (18 %) or high (9 %) risk. Moderately accessible markets were more commonly at low risk (43 %) than at moderate (36 %) or high (21 %) risk.

### Retail prices and percent mark-ups

The median retail price of one AETD varied across anti-malarial category, formulation and outlet type (Table 2). In tablet form, ACT was sold at a median price of US$1.18, compared to US$0.41 for oral non-artemisinin products and US$3.62 for oral AMT. Mark-ups varied across commercial provider types and anti-malarial drugs: the median percent mark-up for ACT ranged between 29 % at village shops and 50 % at drug shops and mobile providers; on oral AMT between 15 % at pharmacies/clinical pharmacies and 60 % at village shops; and on oral non-artemisinin monotherapies between 54 % at pharmacies/clinical pharmacies and 367 % at village shops (Table 3).

**Table 2:** Retail prices of one adult equivalent treatment dose (US$)

Anti-malarial categories formulation		Retailer categories					
		All N = 382	Pharmacies/ Clinical pharmacies N = 77	Drug stores N = 75	Mobile providers N = 101	Grocery stores N = 57	Village shops N = 72
ACT	(n)	(454)	(130)	(101)	(106)	(53)	(64)
All were tablets	Median	1.18	1.18	1.18	1.65	1.11	1.55
	IQR	0.94–1.18	0.71–1.18	0.94–1.18	1.18–2.15	0.71–1.65	1.06–2.12
AMT	(n)	(186)	(34)	(40)	(46)	(29)	(37)
All	Median	4.52	3.61	4.52	4.52	3.61	4.52
	IQR	3.01–12.71	2.44–3.62	2.82–4.52	3.39–22.60	3.01–4.52	3.62–4.52
AMT	(n)	(129)	(15)	(31)	(26)	(25)	(32)
Tablet	Median	3.62	2.64	3.16	3.77	3.62	4.52
	IQR	2.64–3.61	0.00–2.64	2.64–3.16	2.26–4.52	3.01–4.52	3.62–4.52
AMT	(n)	(57)	(19)	(9)	(20)	(4)	(5)
Injectable	Median	22.60	15.10	19.80	22.60	22.60	28.25
	IQR	15.06–26.36	11.30–15.10	18.83–19.77	16.94–28.24	18.83–22.60	28.25–28.25
nAMT	(n)	(88)	(22)	(16)	(22)	(13)	(15)
All	Median	0.46	0.23	0.23	7.41	0.46	1.18
	IQR	0.23–7.41	0.08–0.23	0.23–0.23	0.46–9.88	0.34–0.46	0.46–11.86
nAMT	(n)	(68)	(22)	(13)	(10)	(10)	(13)
Tablet	Median	0.41	0.23	0.23	0.46	0.46	0.68
	IQR	0.23–0.41	0.08–0.23	0.23–0.23	3.41–0.46	0.23–0.46	0.35–11.86
nAMT	(n)	(20)	(0)	(3)	(12)	(3)	(2)
Injectable	Median	9.89	-	14.83	9.89	5.93	17.30
	IQR	7.41–14.83		14.83–14.83	7.41–12.36	3.95–12.36	9.88–17.30

ACT is artemisinin combination therapy; AMT is artemisinin monotherapy; nAMT is non-artemisinin monotherapy; (n) is number of product observations; N is number of retailers for whom information was available; IQR is inter-quartile range. “-“drug category not stocked

**Table 3:** Retail percent mark-ups on one adult equivalent treatment dose (%)

Anti-malarial categories formulation		Retailer categories					
		All N = 382	Pharmacies/ Clinical pharmacies N = 77	Drug stores N = 75	Mobile providers N = 101	Grocery stores N = 57	Village shops N = 72
ACT	(n)	(418)	(119)	(85)	(103)	(51)	(59)
all were tablets	Median	42.8	40.0	50.0	50.0	40.0	28.6
	IQR	20.0–75.0	20.0–80.0	25.0–106.9	25.0–66.7	20.0–60.0	14.3–55.6
AMT	(n)	(177)	(32)	(37)	(46)	(29)	(33)
All	Median	37.1	16.7	29.2	42.9	33.3	60.0
	IQR	20.0–66.7	7.1–42.9	16.7–55.5	25.0–71.4	20.0–41.2	42.9–84.6
AMT	(n)	(121)	(13)	(28)	(26)	(25)	(29)
Tablet	Median	37.1	15.4	37.1	33.3	33.3	60.0
	IQR	20.0–71.4	7.7–25.9	16.7–90.5	25.0–66.7	20.0–41.2	37.1–87.5
AMT	(n)	(56)	(19)	(9)	(20)	(4)	(4)
Injectable	Median	40.0	28.0	29.0	50.0	38.5	47.1
	IQR	19.0–55.6	7.1–42.9	16.7–41.2	25.0–77.8	20.0–42.9	42.9–66.7
nAMT	(n)	(84)	(18)	(16)	(22)	(13)	(15)
All	Median	100.0	53.8	100.0	127.3	177.8	100.0
	IQR	50.0–185.7	7.1–100.0	66.7–150.0	50.0–185.7	33.3–185.7	66.7–150.0
nAMT	(n)	(64)	(18)	(13)	(10)	(10)	(13)
Tablet	Median	100.0	53.8	100.0	100.0	185.7	366.7
	IQR	50–185.7	7.1–100.0	66.7–115.5	80–233.3	33.3–185.7	66.7–366.7
nAMT	(n)	(20)	-	(3)	(12)	(3)	(2)
Injectable	Median	166.7	-	191.7	150.0	33.3	366.7
	IQR	50.0–200.0	-	191.7–275.0	50.0–185.7	17.6–177.8	66.7–366.7

ACT is artemisinin combination therapy; AMT is artemisinin monotherapy; nAMT is non-artemisinin monotherapy; (n) is number of product observations; N is number of retailers for whom information was available; IQR is inter-quartile range. “-“drug category not stocked

### Determinants of retail percent mark-ups

Model 1 had an adjusted R^2^ of 0.144 (Table 4). Percent mark-ups were significantly affected by anti-malarial generic type and brand status, generally reflecting higher percent mark-ups on cheaper products. Mark-ups on the AMT artesunate and non-artemisinin product chloroquine were 60 % (95 % CI 0.45–0.79) and 197 % (95 % CI 1.40–2.77) of the ACT mark-up, respectively. Mark-ups on branded anti-malarials were 66 % (95 % CI 0.51–0.99) of the mark-up on unbranded products. Other product, outlet and market characteristics, including the HHI, had no significant influence on percent mark-ups. Model 2 (the interacted model) had an adjusted R^2^ of 0.168 (Table 4). The effect of the interaction between HHI and malaria transmission risk was not statistically significant (adjusted Wald test p = 0.300) so the term was excluded from the model. Percent mark-ups were significantly affected by market concentration in markets of different levels of accessibility. In remote areas, an increase of the HHI by 0.1 was associated with lower mark-ups (53 %, 95 % CI 0.33–0.85). By contrast, in moderately accessible areas, an increase of the HHI by 0.1 was associated with higher mark-ups (280 %, 95 % CI 1.50–5.25). As observed in Model 1, percent mark-ups were also significantly affected by anti-malarial generic type and brand status.

**Table 4:** Relationship between percent mark-ups and market, outlet and product characteristics (N = 640 anti-malarials)

	Model 1		Model 2	
Variables	Regression coefficient	95 % Confidence-interval	Regression coefficient	95 % Confidence-interval
Market characteristics				
Concentration_HHI volumes	0.79	(0.54–1.16)	0.53***	(0.33–0.85)
At high malaria risk (reference)	-		-	
At moderate malaria risk	0.80	(0.61–1.05)	0.78*	(0.59–1.03)
At low malaria risk	0.88	(0.67–1.16)	0.89	(0.67–1.19)
Remote (reference)	-		-	
Moderately accessible	1.01	(0.72–1.42)	0.50**	(0.29–0.88)
Highly accessible	0.88	(0.68–1.13)	0.85	(0.55–1.34)
Concentration_HHI X Remote (reference)			-	
Concentration_HHI X Moderately accessible			5.58***	(2.10–14.88)
Concentration_HHI X Highly accessible			0.84	(0.35–2.03)
Outlet characteristics				
Located in areas without drug resistance (reference)	-		-	
Located in areas with suspected/confirmed drug resistance	0.97	(0.74–1.27)	1.00	(0.77–1.30)
Pharmacies/Clinical Pharmacies (reference)	-	-	-	
Drug Shop	1.17	(0.86–1.60)	1.14	(0.84–1.54)
Mobile Provider	1.14	(0.84–1.54)	1.02	(0.76–1.38)
Grocery Store	0.90	(0.64–1.27)	0.87	(0.63–1.21)
Village Shop	1.15	(0.75–1.75)	1.04	(0.68–1.60)
Suppliers deliver	1.05	(0.85–1.30)	1.07	(0.87–1.31)
Number of years in operation (year)	0.99*	(0.98–1.00)	0.99*	(0.98–1.00)
Number of AETD sold in past 1 week	0.99*	(0.98–1.00)	0.99*	(0.98–1.00)
Product characteristics				
Tablet (reference)	-		-	
Injectable	1.21	(0.72–2.01)	1.22	(0.73–2.05)
Not branded (reference)	-		-	
Branded	0.66***	(0.51–0.99)	0.66***	(0.51–0.85)
Artesunate + Mefloquine (reference)	-		-	
Artemether	0.71	(0.40–1.28)	0.69	(0.38–1.26)
Artesunate	0.60***	(0.45–0.79)	0.61***	(0.45–0.80)
Chloroquine	1.97***	(1.40–2.77)	2.03***	(1.43–2.86)
Dihydroartemisinin	0.52	(0.11–2.53)	0.54	(0.13–2.34)
Dihydroartemisinin + Piperaquine	1.07	(0.64–1.79)	1.11	(0.65–1.88)
Mefloquine	0.68	(0.43–1.08)	0.67	(0.42–1.06)
Quinine	1.26	(0.68–2.34)	1.25	(0.67–2.32)
Sulfadoxine-Pyrimethamine	1.32	(0.86–2.03)	1.23	(0.79–1.92)
Dihydroartemisinin + Piperaquine + Primaquine	1.38	(0.88–2.16)	1.51	(0.96–2.36)
Constant	84.77***	(49.40–146.94)	108.85***	(62.18–188.67)
Observations	640			
Adjusted R-squared	0.144		0.168	

Coefficients presented in this table are the back-transformed coefficients from the log-linear model. Coefficients were back-transformed by calculating their exponent. The statistical significance of the back-transformed coefficients is therefore inferred by whether 1 is excluded in the 95 % confidence interval. ***p < 0.01, **p < 0.05, *p < 0.1; − is the reference group

## Discussion

This is the first study to investigate the market for malaria treatment in Cambodia using an economic lens, by exploring the determinants of commercial providers’ pricing decisions for anti-malarial drugs. For the interpretation of the study results, a few issues should be taken into account. The analysis of competition requires the difficult task of defining markets on product and geographic dimensions. Outlet census and drug audit data were used to set the product definition as all anti-malarial drug types stocked by all outlets selling anti-malarials. Using the administrative boundary approach, the geographical definition was set as the commune. Ideally household data on treatment seeking behaviour (location and type of provider visited and drugs obtained) would be used for defining the product and geographic definition of the markets, but these data were not available at the time of our study. The definition of markets was therefore validated through qualitative interviews with commercial providers during which information on the provenance of their customers and location of competitors were collected. It is possible that retailers overestimated the degree of competition they faced, as has been observed elsewhere [32], notably from government providers, and that the geographical size of markets was overestimated in the study. However, in the context of Cambodia, where mobile providers represented an important source of anti-malarial drugs [20], it is most likely that a narrower definition of the market boundaries, such as the village, could have underestimated the size of retail markets.

Market concentration measures included both private and public anti-malarial sales, with all government outlets within each market treated as one provider on the basis that government providers were not expected to compete with one another. It is possible that this analytical approach distorted market concentration measures that were later used in the analysis of retail mark-ups, by masking the impact of the relative importance of private sales volumes on private retailers’ mark-ups. This could have been one reason for the difference in the nature of the relationship between mark-ups and concentration in markets at different accessibility levels. However, qualitative interviews with commercial providers highlighted the competitive pressure from VMWs in areas where they operated. Therefore, it was deemed important to include the volumes dispensed at government outlets in the analysis of market concentration as excluding them might have underrepresented the degree of retail competition. This analytical approach was also validated by estimating a model of mark-ups excluding government sales and found the same results as in the models presented here. Differences in wealth across markets may also have influenced retailers’ pricing decisions as, for example, they may have charged higher prices in settings where customers had greater willingness-to-pay. Data on household socio-economic status were not available for these markets at the time of analysis, but the accessibility measure may to some degree have captured this as the accessibility of markets would be expected to be positively associated with household wealth.

It is possible that commercial providers underreported stocking of banned anti-malarial drugs such as AMT, or deliberately misrepresented their pricing behaviour, perhaps by reporting lower selling prices, as they would not want to be seen as making excessive profits, which would have led to an underestimation of mark-ups. Also, the percent mark-ups measured gross margins, which include other costs of selling, and therefore higher mark-ups may not have been a sign of higher net profit margins.

Finally, commercial providers’ price setting behaviour and the extent of price competition in retail markets were analysed by following the traditional SCP approach, investigating whether more concentrated markets were associated with higher mark-ups. Limitations of this approach that hypothesizes a causal link between structure and conduct, combined with that of using HHI as the measure of market competition, are widely recognized in the industrial organization literature [39, 40]. Market concentration as measured by the HHI may be endogenous, that is the result of providers’ pricing decision, demand or cost factors [30, 41]. For example, firms that can produce at a lower cost have the ability to charge lower prices and are therefore likely to have higher market shares than firms with higher costs.

The study assessed the relationship between percent mark-ups for anti-malarials, market concentration measured by the HHI and a set of other market, product and outlet characteristics. In Model 1, no evidence of a significant statistical relationship between anti-malarial mark-ups and the HHI was found, which from a traditional economic theory perspective was surprising. Given the heterogeneity of the markets under study in terms of concentration, the relationship between anti-malarial mark-ups and the HHI in markets at different accessibility and malaria transmission risk levels was explored in Model 2. The expected positive association between HHI and mark-ups was observed in moderately accessible markets only. In these markets, a higher concentration may have indicated less intense competition, offering commercial providers the opportunity to charge higher mark-ups. By contrast, in remote markets an increase in the HHI led to lower mark-ups. One explanation for this difference may be that market concentration was not capturing variations in competitive pressure. With most (82 %) remote markets experiencing moderate or high risk of malaria transmission, lower mark-ups in more concentrated markets may have indicated the competitive pressure of VMWs’ supply of free treatment in areas where they operated, leading private providers to limit their pricing. It is also plausible that retailers operating in hard-to-reach, less populated communes at higher risk of malaria transmission may have put greater emphasis on socially oriented objectives relative to profit maximization. Higher mark-ups in less concentrated remote markets may also have reflected higher costs, for example the costs of transporting anti-malarial supplies. Overall, whilst the price of ACT was set above the recommended retail price, market concentration and mark-ups were not positively associated in most markets. Inadequate ACT coverage for malaria treatment in the private sector was therefore unlikely to primarily reflect excessive retail mark-ups on ACT, and instead reflect other key elements of medicine demand and supply.

Even at a subsidized price, ACT wholesale prices were much higher than those of non-artemisinin monotherapies [21], meaning that even with relatively competitive mark-ups, ACT retail prices were more than two times higher, and therefore may have been beyond the reach of some consumers. Furthermore, while AMT were sold at substantially higher prices than ACT, it may be common for consumers to purchase incomplete doses of AMT as observed elsewhere [42], implying that the price of one adult equivalent treatment dose did not reflect what consumers usually paid [20]. The negative externality of increased pressure for the development of drug resistance caused by AMT use, particularly at low doses, would not be considered by consumers. Also, as noted in the introduction, purchase of drug “cocktails” is common in Cambodia for treating illnesses, including malaria and fevers in general [23]. Although these mixtures of tablets may not contain any anti-malarial, they are often seen as more effective than pre-packaged medicines at treating multiple symptoms [23] and may be perceived by consumers as a substitute for ACT. Overall, it is likely that information on the relative efficacy of ACT was far from perfect among consumers.

The study results indicate that provider pricing may have played an important role in the performance of the market for malaria treatment in Cambodia. At the time of the study, the subsidized ACT wholesale price was US$0.42, which was much higher than the wholesale price of chloroquine (US$0.08), the most popular monotherapy [20, 21]. There may therefore be potential to decrease ACT wholesale and retail prices by applying larger subsidies, such as those provided through the Affordable Medicines Facility – malaria (AMFm). The AMFm implemented in seven African countries made ACT available at a highly subsidized price at the manufacturer level [43], representing 1–20 % of the ex-factory prices [44]. After the introduction of the AMFm, large increases in ACT availability and large decreases in ACT median retail prices were observed in the private sector of most, though not all participating countries [44]. The pooled purchasing power within the AMFm programme also facilitated price negotiations to reduce manufacturer ACT prices [45]. Cambodia was initially included in the group of countries eligible to participate in the Phase 1 of the AMFm pilot but later excluded due to the lack of a first line drug complying with the quality assurance policy of the Global Fund to Fight AIDS, Tuberculosis and Malaria [46]. With a quality approved drug now available for Cambodia, an AMFm-like subsidy may have the potential to decrease wholesale and retail prices as observed in other settings.

Experiences in using RRPs on anti-malarial drugs have proved to have different effects on consumer prices across settings [47–49]. In Cambodia, since the start of the national programme in 2001, the subsidized ACT always had a printed RRP though retail prices generally exceeded it [24, 47]. Little research has been conducted in Cambodia on the level at which RRP should be set. At the time of our study, the RRP for ACT was US$ 0.61, based on a study on consumers’ willingness to pay conducted some years previously [50]. During qualitative interviews conducted in 2009, several private retailers argued that the RRP was set too low and did not provide sufficient profits [21]. Generating evidence on commercial providers’ overhead costs including transport, rent, staff, etc. and their relative importance should be considered when investigating the levels of profit perceived to be “sufficient” by private providers. However, during qualitative interviews, some retailers in Cambodia reported that when consumers were informed about the RRP, they were constrained to sell the subsidized ACT at that recommended price [21], supporting the use of RRP combined with effective communications to consumers. A similar observation can be made from the experiences of countries included in the AMFm pilot. In the three countries in which timely communication campaigns about the AMFm subsidy and RRP were implemented, including Kenya, the Republic of Tanzania (mainland and Zanzibar) and Ghana, median ACT prices were at the RRP levels. By contrast, in countries where promotion activities were delayed or less intense, the median ACT price was above the RRP [49].

Even with lower wholesale prices, retailers may be reluctant to stock ACT if they expect consumer demand to be low because of lack of information on what constitutes appropriate quality malaria treatment. Expanding and intensifying social marketing-like activities, including road shows, radio messages and TV ads combined with increased communication on the regulatory framework could improve consumer information on the need for confirmed diagnosis prior treatment, ACT efficacy compared to older anti-malarial drugs, the potential dangers of cocktail therapies and the negative social externalities of AMT in terms of drug resistance. These activities could also be expanded to non-malarial areas in the context of mobile populations with no or little immunity to malaria who travel to forested areas. Finally, active and supportive regulation has a key role to play in improving the performance of the market for malaria treatment, through measures to stop the importation and distribution of oral AMT and the marketing of cocktail therapies, and through supportive and educative activities with private providers of malaria treatment.

## Conclusion

This study investigates the factors influencing pricing decisions for anti-malarial drugs in Cambodia and contributes to the limited literature on the functioning of health markets in low income countries. Percent mark-ups were significantly affected by anti-malarial characteristics, including generic type and brand status, generally reflecting higher mark-ups on cheaper products. They were also influenced by retail market concentration, although differently in markets at different levels of accessibility, pointing to the heterogeneity of anti-malarial markets in terms of structural characteristics. Key elements of anti-malarial supply and demand, including wholesale pricing and consumer information were however likely to play a bigger role in explaining the limited access to appropriate malaria treatment in Cambodia. The potential for decreasing ACT prices at different levels of the distribution chain by applying a larger subsidy, notably at the manufacturer level, combined with intensive promotion activities towards consumers and strict but supportive regulatory measures should be explored for improving access to appropriate malaria treatment in the private commercial sector in Cambodia.

## Additional file

Additional file 1:
**Correlation Coefficients.** The data provided show the correlation coefficients between the variables used in the models of percent mark-ups.
