# Supplementary material for: Determinants of price setting decisions on anti-malarial drugs at retail shops in Cambodia
Source: Malar J. 2015 May 30;14:224. doi: 10.1186/s12936-015-0737-9 (PMC4462075; doi:10.1186/s12936-015-0737-9)
Supplement: Additional file 1: — Correlation Coefficients. The data provided show the correlation coefficients between the variables used in the models of percent mark-ups. [file 12936_2015_737_MOESM1_ESM.docx]

**Additional file 1: Correlation coefficients**

|  | Concentration (HHI volumes) | Stratum | Accessibility | Malaria risk | AM dosage form | Outlet type | Generic type | Wholesale supplies | Outlet length of operation | AM Brand status | Outlet’s sales volumes |
| --- | --- | --- | --- | --- | --- | --- | --- | --- | --- | --- | --- |
| Concentration (HHI volumes) | 1.00 |  |  |  |  |  |  |  |  |  |  |
| Stratum | 0.13* | 1.00 |  |  |  |  |  |  |  |  |  |
| Accessibility | -0.25* | 0.11* | 1.00 |  |  |  |  |  |  |  |  |
| Malaria risk | -0.04 | 0.25* | -0.01 | 1.00 |  |  |  |  |  |  |  |
| AM dosage form | -0.05 | -0.05 | 0.01 | 0.06 | 1.00 |  |  |  |  |  |  |
| Outlet type | 0.13* | -0.26* | -0.17* | -0.12* | 0.04 | 1.00 |  |  |  |  |  |
| Generic type | -0.05 | -0.08- | -0.01 | -0.17* | -0.14* | 0.14* | 1.00 |  |  |  |  |
| Wholesale supplies | -0.09 | 0.23* | 0.12* | 0.25* | 0.03 | -0.45* | -0.18 | 1.00 |  |  |  |
| Outlet length of operation | -0.09* | -0.03 | 0.13* | -0.03 | 0.03 | 0.02 | 0.01 | -0.01 | 1.00 |  |  |
| AM Brand status | -0.01 | 0.13* | 0.04* | 0.11* | -0.24* | -0.13* | -0.16 | 0.08* | -0.02 | 1.00 |  |
| Outlet’s sales volumes | -0.07 | 0.09* | 0.04 | -0.05 | -0.10 | -0.15 | 0.05 | 0.14* | 0.06 | -0.03 | 1.00 |
